# Dissecting the Profile of Corneal Thickness With Keratoconus Progression Based on Anterior Segment Optical Coherence Tomography

**DOI:** 10.3389/fnins.2021.804273

**Published:** 2022-01-31

**Authors:** Yanling Dong, Dongfang Li, Zhen Guo, Yang Liu, Ping Lin, Bin Lv, Chuanfeng Lv, Guotong Xie, Lixin Xie

**Affiliations:** ^1^Qingdao Eye Hospital of Shandong First Medical University, Qingdao, China; ^2^State Key Laboratory Cultivation Base, Shandong Provincial Key Laboratory of Ophthalmology, Eye Institute of Shandong First Medical University, Qingdao, China; ^3^Ping An Technology (Shenzhen) Co. Ltd., Shenzhen, China; ^4^Ping An Health Cloud Co. Ltd., Shenzhen, China; ^5^Ping An International Smart City Technology Co. Ltd., Shenzhen, China

**Keywords:** keratoconus, corneal thickness, anterior segment optical coherence tomography, deep learning, segmentation

## Abstract

**Purpose:**

To characterize the corneal and epithelial thickness at different stages of keratoconus (KC), using a deep learning based corneal segmentation algorithm for anterior segment optical coherence tomography (AS-OCT).

**Methods:**

An AS-OCT dataset was constructed in this study with 1,430 images from 715 eyes, which included 118 normal eyes, 134 mild KC, 239 moderate KC, 153 severe KC, and 71 scarring KC. A deep learning based corneal segmentation algorithm was applied to isolate the epithelial and corneal tissues from the background. Based on the segmentation results, the thickness of epithelial and corneal tissues was automatically measured in the center 6 mm area. One-way ANOVA and linear regression were performed in 20 equally divided zones to explore the trend of the thickness changes at different locations with the KC progression. The 95% confidence intervals (CI) of epithelial thickness and corneal thickness in a specific zone were calculated to reveal the difference of thickness distribution among different groups.

**Results:**

Our data showed that the deep learning based corneal segmentation algorithm can achieve accurate tissue segmentation and the error range of measured thickness was less than 4 μm between our method and the results from clinical experts, which is approximately one image pixel. Statistical analyses revealed significant corneal thickness differences in all the divided zones (*P* < 0.05). The entire corneal thickness grew gradually thinner with the progression of the KC, and their trends were more pronounced around the pupil center with a slight shift toward the temporal and inferior side. Especially the epithelial thicknesses were thinner gradually from a normal eye to severe KC. Due to the formation of the corneal scarring, epithelial thickness had irregular fluctuations in the scarring KC.

**Conclusion:**

Our study demonstrates that our deep learning method based on AS-OCT images could accurately delineate the corneal tissues and further successfully characterize the epithelial and corneal thickness changes at different stages of the KC progression.

## Introduction

Keratoconus (KC) is a non-inflammatory, chronic, and progressive corneal disease which is characterized by apical thinning and cone-like protrusion of the central cornea, and usually leads to irregular astigmatism and myopia (Kennedy et al., 1986; Hashemi et al., 2020). Reports have shown an incidence of KC to be as high as 1.38/1,000 in the general population (Hashemi et al., 2020). Whereas diagnostic criteria such as CLEK guidelines (Zadnik et al., 1998) and Amsler–Krumeich classification (Krumeich et al., 1998) have been used to grade the severity of KC, the profiles of the corneal thickness along with KC progression are yet to be defined. Corneal thickness including epithelial thickness has been considered as one of the most important morphological features that aids in the characterization of KC progression (Li et al., 2012; Xu et al., 2016; Morishige et al., 2019; Yang et al., 2020; Toprak et al., 2021). Thus, characterizing the corneal thickness at different stages of KC might complement the existing diagnostic criteria.

With its ability for high-resolution non-invasive imaging in cross-sectional biological systems, anterior segment optical coherence tomography (AS-OCT) is an effective tool in observing the whole corneal thickness as well as individual layers such as the epithelium in normal or KC eyes (Chen et al., 2012; Corre-Perez et al., 2012; Li et al., 2012; Xu et al., 2016; Ang et al., 2018; Morishige et al., 2019; Yang et al., 2020; Toprak et al., 2021). Compared with the normal eyes, KC eyes have thinner apical corneal epithelial thickness but thicker epithelial layer superonasally, which is similar to the total corneal thickness pattern (Li et al., 2012). Eyes with forme fruste keratoconus seem to have increased central epithelium/stroma ratio and asymmetric superior-nasal epithelial thinning (Toprak et al., 2021). Besides, some epithelial thickness-based variables and corneal thickness-based variables have been developed for detecting KC (Li et al., 2012; Yang et al., 2020; Toprak et al., 2021). With ultra-high-resolution OCT, vertical thickness profiles of the epithelial and Bowman’s layers have been shown to provide valuable diagnostic references for sub-clinical KC (Xu et al., 2016). Recent studies have investigated corneal deformation with the presence of stromal scarring in KC patients and demonstrated a correlation between the progression of KC and a reduction in corneal thickness and volume, as well as stromal scar formation (Morishige et al., 2019). These studies provided useful insights into the potential use of corneal thickness in understanding underlying mechanisms of KC. However, there is no study on the quantification of the characteristics of corneal and epithelial thickness at the different stages of KC development (Zadnik et al., 1998; Morishige et al., 2019).

One of the important premises for obtaining corneal thickness is accurate segmentation of corneal tissue interfaces from the AS-OCT images. Currently, the corneal tissue segmentation often performed through either manual labeling or some traditional image processing algorithms (Larocca et al., 2011; Li et al., 2012; Xu et al., 2016; Ang et al., 2018; Morishige et al., 2019; Yang et al., 2020; Toprak et al., 2021). Whereas manual labeling is time-consuming and has poor repeatability, the traditional image processing methods are less robust to deal with pathological corneas (Larocca et al., 2011; Williams et al., 2015; Ang et al., 2018; Elsawy et al., 2019). Recent studies have explored the feasibility of using deep learning-based methods for corneal tissue segmentation with AS-OCT images (Mathai et al., 2019; Ouyang et al., 2019; Santos et al., 2019). We have also proposed a hierarchy-constrained network, which robustly improves the segmentation performance of the corneal tissue interfaces in both normal and KC eyes (Liu et al., 2020). By taking advantage of this automated method, the profiles of the corneal thickness could be conveniently determined from the AS-OCT images.

In this study, we aimed to investigate the corneal and epithelial thickness profiles along the vertical and horizontal meridians in the KC eyes at different stages. The corneal tissue interfaces were delineated using our previously developed hierarchy-constrained method (Liu et al., 2020), and the thickness was automatically measured from the segmented AS-OCT images. Then one-way ANOVA and linear regression analyses were performed to explore the trends of the thickness changes against the KC progression.

## Materials and Methods

### Dataset

This retrospective study was conducted based on the tenets of the Declaration of Helsinki and was approved by the institutional review board of Qingdao Eye Hospital of Shandong First Medical University. A total of 1,430 images from 715 eyes were selected from a large clinical database of the hospital between January 2009 and July 2021. The 715 eyes included both normal and KC patients. We excluded participants with any type of prior ocular surgery or trauma, associated corneal pathologic features, and those who had undergone collagen cross-linking, corneal rings, or keratoplasty. All AS-OCT images were acquired by Optovue RTVUE 100 (Optovue, Inc., United States), using a line scan mode. Each eye acquired two scans along with the horizontal and vertical meridians. The pupil center was treated as the focus point during scanning. The acquired images had a resolution of 1,019 × 640 pixels and covered an area of 8 mm × 1.933 mm. All the images were unified to 1,024 × 640 pixels by padding zeros on the left and right sides, and then resizing to 2,648 × 640 pixels for isotropy. Consequently, each pixel represented approximately 3 μm in corneal tissue, both horizontally and vertically.

The KC eyes were categorized into four different stages based on both CLEK guidelines (Zadnik et al., 1998) and additional clinical criteria (Morishige et al., 2019). According to the CLEK guidelines (Zadnik et al., 1998), we first identified three stages including mild KC (corneal curvature is less than 45 D), moderate KC (corneal curvature is between 45 D and 52 D), and severe KC (corneal curvature is more than 52 D). Then the scarring KC was classified based on the existence of stromal scarring (Morishige et al., 2019). All patients with scarring have resolved hydrops. In particular, the cornea, which appears as Descemet’s membrane rupture with dilacerations of collagen lamellae, large fluid-filled intra-stromal cysts, was excluded from the scarring stage. In total, there were 118 normal eyes, 134 mild KC eyes, 239 moderate KC eyes, 153 severe KC eyes, and 71 scarring KC eyes. The demographic information is shown in Table 1.

**TABLE 1:** Demographic information for five groups in KCTD.

	Normal	Mild KC	Moderate KC	Severe KC	Scarring KC
Eyes	118	134	239	153	71
Images	236	268	478	306	142
Sex (M:F)	93:25	112:22	178:61	129:24	58:13
Age (mean ± std)	24.39 ± 6.69	21.01 ± 4.87	20.62 ± 4.43	22.58 ± 5.91	20.73 ± 5.54

*KC, Keratoconus; KCTD, KC corneal thickness dataset.*

### Deep Learning Based Corneal Segmentation

To measure the corneal layers’ thickness, we first performed corneal tissue segmentation using our proposed hierarchy-constrained network (Liu et al., 2020). The network adopted the U-Net architecture (Ronneberger et al., 2015) and consisted of a progressive feature-extraction module (PFEM) and a multi-level prediction fusion module (MPFM) (Liu et al., 2020). The PFEM added side paths to each level of decoder to achieve deep supervision for obtaining correct image features. On the other hand, the MPFM leveraged semantic information in various resolutions by concatenating reconstructed features from each level of the decoder. In addition, we extracted the boundaries of layers to calculate edge loss as additional constraints. Our previous report has shown that such a deep learning-based method improves the performance of corneal tissue segmentation (Liu et al., 2020). The main code is available at https://github.com/sie163/ASOCT_KC.

Before applying the segmentation method to the entire dataset, we evaluated its accuracy on a partial subset. Specifically, 236 normal images (from 118 normal eyes) were numbered, and 150 numbers were randomly generated from 1 to 236 and the images corresponding to the numbers were extracted to form a subset. The same rule was applied to KC images. Finally, we randomly selected 150 normal images and 160 KC images from the AS-OCT dataset, and manually labeled the semantic masks. The boundaries of the cornea and epithelial layer were outlined with customized labeling software by 3 ophthalmologists. Before formal labeling, we verified the labeling consistency of 3 ophthalmologists on 10 AS-OCT images covering different KC stages. Paired comparison of the labeled results showed that the dice values of corneal segmentation all reached 0.99 and the dice values of epithelial segmentation all reached 0.95. It showed that the labels of 3 ophthalmologists were basically the same. In addition, all 310 images were labeled, about 1/3 for each ophthalmologist. Thereafter, a senior expert reviewed the labeled semantic masks and discussed with 3 ophthalmologists to revise the questionable masks. Finally, all 310 AS-OCT images with labeled masks were used to evaluate the performance of our proposed deep learning method.

### Measurement of the Corneal Thickness

**FIGURE 1:**
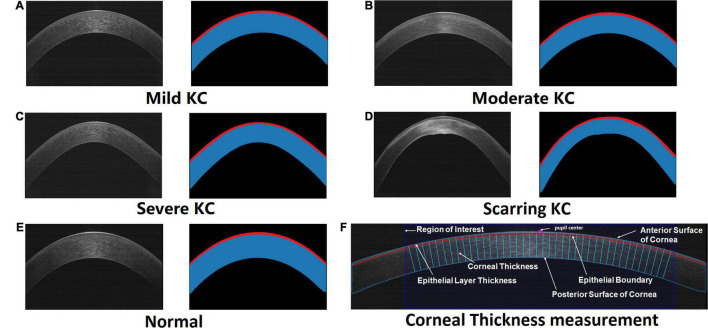
The original AS-OCT images and their corresponding segmented maps generated by our trained deep learning networks. Red masks illustrate the segmented epithelial layers, while masks with red and blue are the segmented corneas. The representative cases included mild KC **(A)**, moderate KC **(B)**, severe KC **(C)**, scarring KC **(D)**, and normal eye **(E)**, respectively. **(F)** An example of the automatic corneal thickness measurement on resized AS-OCT image. The purple circle denotes the pupil center on the cornea. The dark blue rectangle stands for the studied region of interest. The sky-blue and red curves draw the anterior and posterior boundaries of the cornea including the epithelial layer. The normal lines of the corneal anterior surface represent the measurement of the epithelial layer thickness and corneal thickness.

To automatically measure the corneal thickness, the pupil center in the anterior corneal surface was first defined as a reference point after corneal segmentation. A Region of Interest (ROI) was then derived by cutting off 3 mm sections on either side of the reference point. Thus, an ROI containing 6 mm of the studied corneal section was created. Locating the reference point and corresponding ROI was essential because it serves to map the corneal tissues for further analysis of the corneal layers’ thickness. To calculate the thickness of the cornea and epithelium, we then set 40 sampled points in the ROI, which were measured after every 0.15 mm horizontally or vertically. The distance between the sampled points on the anterior surface of the cornea and the intersection points of its incident normal as well as the boundary of epithelial and posterior surface of the cornea were defined as the thickness of the cornea and epithelial layer, respectively (Figure 1F). The visualization was implemented using Python (Oliphant, 2007).

### Segmentation Evaluation and Statistical Analysis of Corneal Thickness Profiles

The performance of the corneal segmentation was assessed by direct and indirect evaluation metrics. The direct metrics included dice coefficient, IoU, sensitivity, and specificity (Liu et al., 2020), while the indirect metric was only the thickness error. Dice coefficient and IoU represented the overlap between the segmented and the labeled areas by the clinician, which reflects the overall segmentation precision. Sensitivity and specificity were the auxiliary metrics for proper segmentation. Whereas sensitivity calculated the proportion of positive pixels that are correctly segmented, specificity calculated the proportion of negative pixels that are properly segmented. We calculated these metrics for the segmentation of the whole cornea and epithelium. Besides, we measured the corneal and epithelial thickness based on the segmented maps and the labeled maps, respectively, and then calculated the average values of their differences as an indirect evaluation metric.

In addition, the thickness profiles in the horizontal and vertical meridians were compared between the normal eyes and the different KC stages. To unify the direction, the horizontal scan of the right eye was mirrored to the left eye during comparison. Each thickness line was divided equally into 20 zones, and the mean values and 95% confidence intervals (CI) were calculated. We applied one-way ANOVA to investigate group effect for mean epithelial thickness and corneal thickness in each zone. On the other hand, a two-sample *t*-test was used to determine the statistical significance of between-group differences. A *P* < 0.05 was considered as statistically significant. Besides, we applied ordinary least square linear regression to investigate the trend of corneal and epithelial thickness along with the progression of the KC in each zone. To test the diagnostic values of the epithelial and corneal thickness profiles, four thickness ectasia indices, including epithelium ectasia index of the vertical meridian (EEI_V), cornea ectasia index of the vertical meridian (CEI_V), epithelium ectasia index of the horizontal meridian (EEI_H), and cornea ectasia index of the horizontal meridian (CEI_H), were built to quantify the different change patterns at the different KC stages. Thickness ectasia index is defined as the ratio of maximum thickness to minimum thickness. Moreover, then linear discriminant analysis was applied to build discriminant functions with four indices. The predictive accuracies of differentiating the groups with different KC stages from the normal group were determined by receiver operating characteristic (ROC) curves and the area under the curves (AUC). All statistical analyses were performed with SciPy library in Python (Oliphant, 2007).

## Results

### Evaluation of the Corneal Segmentation Algorithm

Our experiment demonstrated successful segmentation of the different stages of the KC and normal eyes (Figure 1). Our designed hierarchy-constrained network enhanced the capability of identifying corneal layers’ boundaries from indiscernible images, such as having some degree of scarring around the boundary as shown in Figure 1D.

The quantitative evaluation of the segmentation performance was performed based on 310 labeled AS-OCT images. Using our model, there was high consistency of the segmented results with tissue masks labeled by clinical experts for both the whole cornea and epithelial layers in normal and KC eyes (Table 2). Besides, whereas dice coefficient, IoU, sensitivity, and specificity were slightly lower in the KC eyes compared with the normal eyes, their values significantly improved to 0.989, 0.978, 0.991, and 0.995 for the whole cornea, and 0.925, 0.860, 0.932, and 0.997 for the epithelium, respectively. The thickness error (T_error) between the segmented and labeled maps was 2.220 μm for the epithelial thickness and 1.788 μm for the corneal thickness in the normal eyes. On the other hand, the T_error between the segmented and labeled maps was 3.858 μm for the epithelial thickness and 3.462 μm for the corneal thickness in the KC eyes. The error range of thickness was, therefore, just about one pixel, because each image pixel represented around 3 μm both horizontally and vertically.

**TABLE 2:** Quantitative evaluation for corneal tissue segmentation and thickness measurement between our model and clinicians.

	Dice	IoU	Sensitivity	Specificity	T_error (μm)
**Epithelium**					
Normal	0.951	0.906	0.954	0.998	2.220
KC	0.925	0.860	0.932	0.997	3.858
**Cornea**					
Normal	0.995	0.990	0.996	0.997	1.788
KC	0.989	0.978	0.991	0.995	3.462

### Statistical Analysis of the Thickness Changes

**FIGURE 2:**
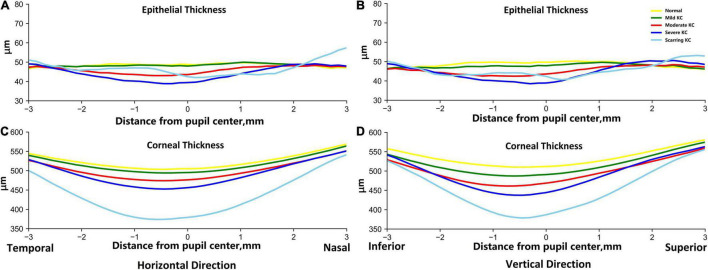
The average epithelial and corneal thickness profiles in the horizontal **(A,C)** and vertical **(B,D)** meridians between the normal and KC eyes in different stages. The reference origin is the pupil center in the scanned AS-OCT image. The horizontal direction is from temporal to nasal, and the vertical direction is from inferior to superior. The yellow lines are for normal groups, and the green, red, dark blue, and sky-blue lines are for mild KC, moderate KC, severe KC, and scarring KC, respectively.

The profiles of the corneal and epithelial thickness in the horizontal and vertical meridians were then investigated based on all 1,430 AS-OCT images (Figure 2). For the epithelial thickness, there were minimal differences and fluctuations between the normal and mild KC eyes both in the horizontal and vertical meridians. With the progressing of the KC, the central epithelial layer grew thinner, thus the severe KC eyes had the thinnest central epithelial thickness. The epithelial thickness was thicker in the scarring KC stage and was accompanied by irregular fluctuations in both the horizontal and the vertical meridians. For the corneal thickness, the average changes of the thickness profiles were very regular, which were characterized by gradual thinning with KC progression. The thinnest parts were in the temporal and the inferior side next to the pupil center for the horizontal and the vertical meridians, respectively.

**FIGURE 3:**
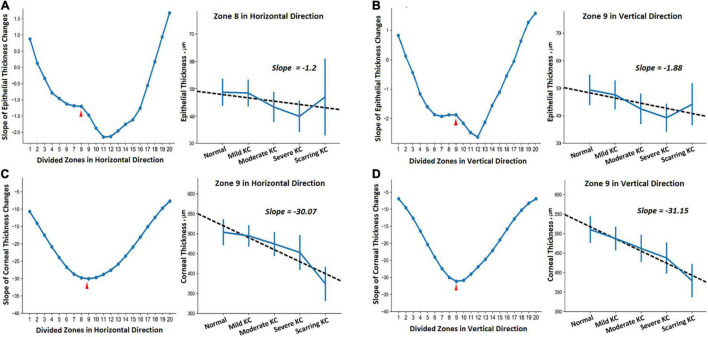
The trends of the thickness change in different zones of the horizontal **(A,C)** and vertical **(B,D)** meridians between the normal and KC eyes in different stages. **(A,B)** Show epithelial thickness changes, while **(C,D)** show the corneal thickness changes. Each subgraph includes the fitted slopes in all divided zones and the detailed regression curve in one of the selected zones.

In addition, one-way ANOVA revealed significant thickness differences in all the divided zones in the horizontal and vertical meridians (*P* < 0.05). We also assessed the trends of thickness changes in each zone in the normal and KC eyes in different stages (Figure 3). The fitted slopes showed similar curve shapes for corneal thickness. The values were all less than zero, which demonstrated that the corneal thickness grew thinner with the KC progression. The lowest slopes occurred at zone 9 both in the horizontal and vertical meridians. However, the slope curves were relatively complex for the epithelial thickness. There were some positive values in the peripheral region of both sides, including zone 1, 2, 18, 19, and 20 for the horizontal meridian, and zone 1, 2, 17, 18, 19, and 20 for the vertical meridian. The lowest slopes occurred at zone 11 and zone 12 for the horizontal and the vertical meridians, respectively. In particular, there were obvious fluctuations around zone 8 for the horizontal meridian and zone 9 for the vertical meridian.

Furthermore, we provided the 95% confidence intervals (CI) for the thickness values (Table 3) and their corresponding regression curves for the typical zones (Figure 3). The selected zones included epithelial thickness in zone 8 of the horizontal meridian (ET_Z8_H) and the corneal thickness in zone 9 of the horizontal meridian (CT_Z9_H) as well as the epithelial thickness (ET_Z9_V) and the corneal thickness (CT_Z9_V) in zone 9 of the vertical meridian. Two sample *t*-tests revealed that most comparisons of the corneal thickness were statistically different among each group in both meridians (CT_Z9_H and CT_Z9_V). Most of the differences had a significance of *P* < 0.001, except between the normal and mild KC eyes in zone 9 of the horizontal meridian (*P* = 0.07). For the epithelial thickness, most comparisons reached a *P* < 0.05. Besides, due to irregular changes of the epithelial thickness in scarring KC, there were no significant differences between the normal eyes and mild KC (*P* = 0.37), or between scarring KC and normal eyes (*P* = 0.28), mild KC (*P* = 0.47), and moderate KC (*P* = 0.08) in zone 8 of the horizontal meridian (ET_Z8_H). In addition, there were no significant differences between scarring and moderate KC (*P* = 0.29) in zone 9 of the vertical meridian (ET_Z9_V).

**TABLE 3:** A 95% CI of epithelial thickness and corneal thickness in selected zones.

Group	ET_Z8_H (μm)	ET_Z9_V (μm)	CT_Z9_H (μm)	CT_Z9_V (μm)
Normal	47.84–49.93	48.37–50.84	497.28–511.68	503.88–519.23
Mild KC	47.19–49.26	46.73–48.72	490.2–501.83	482.10–493.82
Moderate KC	42.87–44.56	41.79–43.30	470.94–480.41	456.58–466.42
Severe KC	39.20–41.47	38.86–40.81	447.38–463.22	432.33–447.57
Scarring KC	43.34–50.46	41.17–47.16	363.07–389.10	369.19–402.14

*CI, confidence intervals; ET_Z8_H, epithelial thickness in zone 8 of horizontal meridian; ET_Z9_V, epithelial thickness in zone 9 of vertical meridian; CT_Z9_H, corneal thickness in zone 9 of horizontal meridian; CT_Z9_V, corneal thickness in zone 9 of vertical meridian.*

**FIGURE 4:**
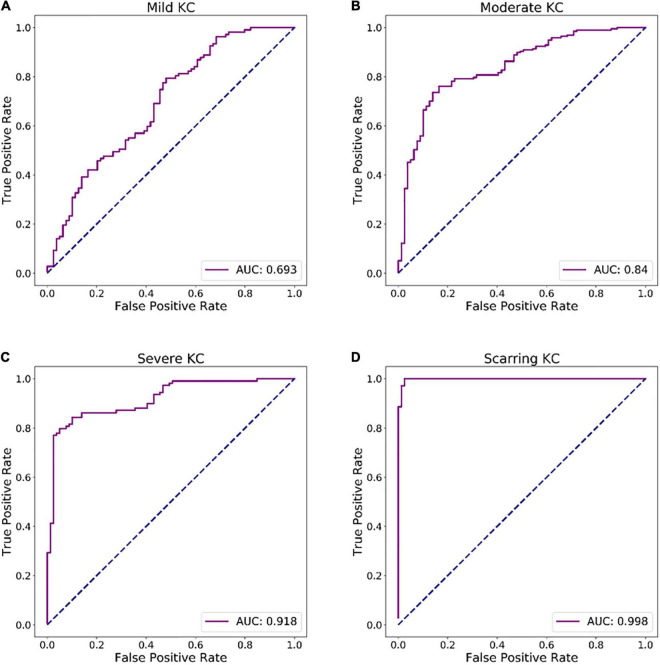
ROC curves of discriminant functions in differentiating the groups with different KC stages from the normal group. **(A)** ROC curve of discriminant function for mild KC vs. the normal group. **(B)** ROC curve of discriminant function for moderate KC vs. the normal group. **(C)** ROC curve of discriminant function for severe KC vs. the normal group. **(D)** ROC curve of a discriminant function for scarring KC vs. the normal group.

In Figure 4, the ROC curves for each discriminant function illustrated the discriminative abilities of mild KC, moderate KC, severe KC, and scarring KC from normal eyes. The output values of the discriminant functions showed different abilities to discriminate mild KC (AUC = 0.693), moderate KC (AUC = 0.840), severe KC (AUC = 0.918), and scarring KC (AUC = 0.998) from normal eyes, respectively. The more severe keratoconus, the higher the diagnostic accuracy.

## Discussion

As described in the global consensus on KC (Hashemi et al., 2020), the existing staging standards of KC [CLEK guidelines (Zadnik et al., 1998) and Amsler–Krumeich classification (Krumeich et al., 1998)] are relatively limited and outdated. For instance, the protocols do not fully consider the variation trend of various parameters such as the anterior and the posterior corneal surface height, the cornea thickness, and cornea curvature (Hashemi et al., 2020). Interestingly, corneal thickness variation is one of the most important characteristics of KC progression. To better guide clinicians in staging of the KC, there is need for accurate measurement of the corneal thickness and analysis of the corneal thickness distribution in patients with KC at different stages. AS-OCT provides cross-sectional information critical in the generation of thickness maps of both the whole cornea and individual layers (Shan et al., 2019; Yip and Chan, 2019). The main obstacle to accurate evaluation of the thickness is precise outlining of the corneal tissue boundaries. Besides the manual segmentation or semi-automated traditional methods (Chen et al., 2012; Corre-Perez et al., 2012; Li et al., 2012; Xu et al., 2016; Ang et al., 2018; Morishige et al., 2019; Yang et al., 2020; Toprak et al., 2021), deep learning-based methods have been proposed for corneal tissue interface segmentation (Mathai et al., 2019; Ouyang et al., 2019; Santos et al., 2019). In our previous studies, we compared these methods with our proposed hierarchy-constrained segmentation network (Liu et al., 2020) and validated the effectiveness of our network architecture and boundary constraint. Here, we further demonstrated that our method could achieve accurate corneal segmentation for measuring corneal and epithelial thickness in both normal and KC eyes. Our experiment demonstrated high segmentation accuracy as evaluated by dice coefficient, IoU, sensitivity, and specificity (Table 2). In addition, the error ranges of the corneal thickness were less than 4 μm between our automatic method and the results by clinical experts, which was just about one image pixel.

Although the corneal thickness profiles including the epithelial layer have been investigated based on AS-OCT images (Chen et al., 2012; Corre-Perez et al., 2012; Li et al., 2012; Wu et al., 2014; Ma et al., 2016; Xu et al., 2016; Ang et al., 2018; Mathai et al., 2019; Morishige et al., 2019; Ouyang et al., 2019; Santos et al., 2019; Wang et al., 2019; Yang et al., 2020; Toprak et al., 2021), none of them was able to successfully classify the KC into different stages based on the CLEK guidelines (Zadnik et al., 1998). Often, all the KC eyes are considered as a group when compared with normal eyes. Only two recent studies classified sub-clinical KC (Xu et al., 2016) and scarring KC (Morishige et al., 2019). In this study, we collected KC eyes at four different stages: mild, moderate, severe, and scarring KC. The thickness profiles were revealed within ± 3 mm from the pupil center along with the horizontal and vertical meridians. For the corneal thickness, the overall distribution showed that the cornea was thinner in the central part but thicker on the peripheral sides. The thinnest points were located around 0.5 mm temporal side (CT_Z9_H) and 0.5 mm inferior side (CT_Z9_V) from the pupil center. The thinning phenomenon in the middle became more pronounced with the progression of the KC. On the other hand, for the epithelial thickness, the biggest change happened in the scarring KC with irregular thinning or thickening. Descemet’s layer might break spontaneously to produce corneal edema in the late stage of the KC. Thereafter, the scar tissues were formed after corneal edema was repaired, which undoubtedly caused the irregular changes of epithelial thickness in the scarring KC. Since the thickness profiles in some stages of KC had never been investigated based on the AS-OCT, the findings not only supported the previous studies (Li et al., 2012; Ma et al., 2016; Xu et al., 2016; Morishige et al., 2019; Wang et al., 2019; Yang et al., 2020; Toprak et al., 2021), but also provided new evidence for the KC features at different stages. Furthermore, our experimental results with the linear discriminant analysis revealed that the measured thickness indices could be used to differentiate the groups of different KC stages from normal eyes.

Some limitations should be considered and need to be strengthened. First, previous studies have investigated the consistency of the corneal thickness measurement with AS-OCT, ultrasound pachymetry, and Scheimpflug imaging (Chen et al., 2012; Dutta et al., 2013; Huang et al., 2013; Kiraly et al., 2017). To prove the effectiveness of our proposed method, there is a need to perform more comparison studies with other devices or methods in the future. Second, besides the CLEK guidelines, there are more relevant KC grading systems such as topographical keratoconus classification (TKC) (Issarti et al., 2019). Further work includes grading the data with the TKC system and applying our purposed method to characterize the thickness changes along with the TKC system. Third, our study only obtained two AS-OCT images along with the horizontal and vertical meridians. A recent study that scanned 16 AS-OCT images for each eye to form a three-dimensional corneal shape (Morishige et al., 2019). Thus, we could scan more intensive images and detect the KC at different stages based on the AS-OCT derived corneal thickness.

Taken together, with our proposed segmentation network, we could successfully quantify the epithelial and corneal thickness profiles in the horizontal and vertical meridians for the normal and KC eyes at different stages. The entire corneal thicknesses became thinner with the progression of the KC, and their trends were deepened especially around the pupil center with a slight shift to the temporal and inferior side. Besides, the epithelial thickness had more irregular fluctuations due to more complex corneal tissue changes in the scarring KC. These findings therefore provide more quantitative information to investigate the underlying mechanism of KC at different stages.

## Data Availability Statement

The original contributions presented in the study are included in the article/supplementary material, further inquiries can be directed to the corresponding authors.

## Ethics Statement

The studies involving human participants were reviewed and approved by the Institutional Review Board of Qingdao Eye Hospital of Shandong First Medical University. Written informed consent to participate in this study was provided by the participants’ legal guardian/next of kin. Written informed consent was obtained from the individual(s), and minor(s)’ legal guardian/next of kin, for the publication of any potentially identifiable images or data included in this article.

## Author Contributions

YD, DL, YL, and BL carried out the entire procedure including the literature search, data extraction, method development, statistical analysis, and writing the manuscript. PL and ZG finished the experimental design and clinical interpretation. YL, BL, and CL oversaw method development, statistical analysis, and revision of the manuscript. GX and LX conceived of the study and revised the manuscript. All authors read and approved the final manuscript.

## Conflict of Interest

YL, BL, CL, and GX were employed by Ping An Technology (Shenzhen) Co. Ltd. The remaining authors declare that the research was conducted in the absence of any commercial or financial relationships that could be construed as a potential conflict of interest.

## Publisher’s Note

All claims expressed in this article are solely those of the authors and do not necessarily represent those of their affiliated organizations, or those of the publisher, the editors and the reviewers. Any product that may be evaluated in this article, or claim that may be made by its manufacturer, is not guaranteed or endorsed by the publisher.
